# The Present and Future of Homœopathy

**Published:** 1883-09

**Authors:** C. Pearson

**Affiliations:** President


### THE PRESENT AND FUTURE OF HOMŒOPATHY.

*An Address Delivered before the International Hahnemannian Association at Niagara Falls, June 19th, 1883.*

BY C. PEARSON, M. D., PRESIDENT.

It is difficult to speak of the future of Homœopathy without referring briefly to the past, and in doing this we cannot go back anterior to 1810, when Hahnemann published the first edition of his *Organon* of the healing art. Prior to this, though the laws and principles forming the base for this grand superstructure had at different times been observed by others, they had never been utilized, or systematized, and hence Homœopathy was unknown. Hahnemann then, being the father, or, in every proper sense, the discoverer of Homœopathy, it becomes very apparent that to practice it at all we must practice what he taught. It seems that during his lifetime a number of physicians, called by him "a new mongrel sect," had adopted a kind of spurious practice, or had engrafted this on to the tree of Homœopathy, where it grew, and with which its branches so intermingled that the parent stem was little thought of, and as the fruit was not extensively known, this hybrid product was palmed off on the market as a genuine article. A few shoots from the original tree were transplanted in this country in the years 1825, 1828, 1836, and 1839, and for a time the fruit showed no signs of deterioration.

But it is well known to horticulturists that to preserve melons in their purity they must not be planted in close proximity to squashes or any other of the *cucurbita* family. And so this tree of Homœopathy had unhealthy surroundings, the atmosphere was bad, and while its growth was rank and vigorous enough, it was dwarfed by an indigenous undergrowth of ignorance, prejudice, and ridicule, in consequence of which the fruit became *bitter*, and in many instances acted on those who used it as a cathartic, or an emetic.

In view of this state of affairs, a society was formed some forty years ago by good men and true to better promote the cultivation and cause of Homœopathy, and for a time it did stalwart work, but many of the old guard no longer answer at roll-call, and it is greatly to be feared that the inheritance they left is not guarded with scrupulous care by their degenerate sons.

Mock-oranges and Dead Sea apples are again in the market, labeled "Homœopathy," and no course of reasoning seems adequate to convince either the vender or consumer that the article is not genuine. It is in vain that the *Organon* is pointed to as the guide and the law; these trades' unions deny that "it is the standard of the Homœopathy of to-day." And if that be true, one or the other must have changed. Which is it? The former was once regarded as the only criterion by which the homœopathicity of any proposition could be tested. Has it changed? No. There are the words of Hahnemann just as he penned them three-fourths of a century ago. But they tell us three-fourths of a century is too long for a medical doctrine to endure, that this is a progressive age, that what is regarded as authority to-day may not be so considered to-morrow. And this is true when applied to systems founded on theory alone, but not true in regard to natural laws. Euclid wrote two thousand years before Hahnemann, and yet his *Elements of Geometry* are text-books to-day. Kepler wrote two hundred years before, and the only improvements made in relation to his remarkable discoveries known as "Kepler's laws" have been in reference to their application. Truth, then, is in all ages the same; it is no better or none the worse of having been discovered in this or in any other age; it is, in short, of all time and for all time, and though newly discovered, it cannot well be said to be new. A diamond will ever remain a diamond, though it may be trodden under foot by the unappreciative.

If then the teaching and so-called homœopathic practice of to-day be not in accord with the *Organon*, it is not difficult to see that what is thrown on the market as Homœopathy is a spurious article with which Hahnemann, were he living, would not have fellowship. Says a writer in the *Medical Investigator* of March 15th, 1882: "The immediate followers of Hahnemann are passing away." How long and how often have we heard this and similar remarks in reference to Homœopathy—good, bad and indifferent? Still, its ranks have been steadily filling up, and when, by fermentation, it has thrown off the foam, impurities, and crudities that attach themselves to it, will its inherent principles, like gold from the quartz, stand out in bold relief.* It is, of course, optional with any one to reject the doctrines of Homœopathy as taught by Hahnemann, but consistency would demand that those who do so should reject its name as well. The members of the International Hahnemannian Association wish to retain both, in practice as well as in theory, and one of the principal objects of its organization was that both might be retained.

In my address last year I endeavored to show what Homœopathy was as taught by its founder, and this it is not necessary to repeat; neither is it necessary before this Association and for your edification to allude to the disease-producing, as well as the disease-curing, effects of microscopic particles of matter; but as it is not the well that need the physician but only the sick, and as the great mass of the human family, both physicians and laymen, are sick so far as a knowledge of Homœopathy is concerned, it may not be considered a digression to briefly refer to some of the evidences of our faith with which the physical world is filled. One of the great popular mistakes seems to be that matter always produces its effects in proportion to its bulk, instead of its affinity for other matter or adaptability to peculiar idiosyncracies. Herbert Spencer says: "A minute portion of a virus introduced into an organism does not work its effects proportionately to its bulk, as would an inorganic agent on an inorganic mass, but by appropriating materials from the blood of the organism and thus immensely increasing, it works

---

* In one of our avowedly homœopathic journals the editor recently said, "Homœopathy is one thing and the teaching of Hahnemann another." The word *modern* before Homœopathy would convert falsehood into truth.

effects altogether out of proportion to its bulk as originally introduced—effects which may continue with accumulating power throughout the remaining life of the organism. This is so with internally evolving agencies, as well as with externally invading agencies. A portion of germinal matter itself microscopic may convey from a parent some constitutional peculiarity that is infinitesimal in relation even to its minute bulk; and from this there may originate fifty years afterward gout or insanity in the resulting man." Grauvogle, who found that ten drops of the tincture of arsenic in thirty quarts of water, corresponding in proportion with the third potency, when taken in tablespoonful doses four times a day, produced on himself much more feeble effects than a few drops of the thirtieth potency of the same remedy in a pint of water repeated in the same manner. And if such is the result in health, what must it be when the nerves and tissues are rendered highly sensitive by inflammation? At the same time, Grauvogle was far from being a spiritualist in medicine, or from adopting the dynamic theory of Hahnemann. He believed that to whatever extent our potencies may be carried, their action in the system is still that of matter on matter. It is unfair, therefore, to regard our mode of preparing potencies as a mere diluting or weakening process. A small portion of yeast under favorable circumstances will convert a large bulk of flour into yeast; a small quantity of this transferred a second, third, or an indefinite number of times in the same way will produce the same results. In a similar manner, a small particle of a medicinal substance will medicate a non-medicinal vehicle, converting the whole into medicine though the process be repeated a thousand times.

As the chlorate of potash has been found in the blood cells in particles smaller than the quadrillionth part of a cubic millimeter, so small, in fact, that it cannot be conceived of by any of our senses, we might conclude that our process of attenuation develops in the drug nothing that could well be called spirit but merely renders the particles of matter finer and more capable of entering these blood cells, producing in them important changes. Liebig found that a concentrated solution of common salt was unsuited to act as a function remedy; that it was necessary to dilute or attenuate it in fifty times its weight of water; and Valentin says that a grain of salt hardly large enough to taste contains billions of groups of atoms, which no mortal eye can ever grasp. From this it would appear that medicines have no other action than that which finely attenuated matter has on other material particles in obedience to chemical or physiological laws. But, it may well be asked, how is it, then, that in less than one hour after taking ten grains of quinine it is found that every particle of it in weight has in the excretions passed out of the system? What then produces the effects of this drug, which we know will last for days, weeks, or even longer? What is the fragrance of a flower, or the power of the magnet, which these bodies throw off without in any perceptible degree effecting a change in the bodies themselves? A grain of musk would impregnate a thousand cubic feet of air for a hundred years and still retain its original weight. The Mosque of Omar was built in the seventh century; the mortar in its walls was impregnated with musk, and though twelve hundred years have come and gone it is said the atmosphere in the building still retains the odor. Other substances more volatile would have in much less time entirely evaporated, but to become invisible even to one of our modern microscopists is not conclusive evidence that in some form they do not exist. Dr. Myerhoffer by aid of the solar microscope claims to have detected particles of platinum and mercury after a grain of these metals had been divided more than a trillion times; lead and iron a billion times; zinc, copper, tin, silver, and gold more than a million. Seguin and Rummel profess by the same means to have seen metallic atoms in the 200th potency. Less than two grains of copper dissolved in nitric acid and diluted with water tinged blue with ammonia can be divided into 50,000,000,000 visible parts. The same amount of carmine may be divided into 2,600,000,000 parts, all equally visible. Professor Jaeger, in his Neural Analysis with the electro-magnetic galvanometer, has detected medicinal force in the 4,000th centesimal potency; but it remains for the still more sensitive diseased human organism to respond to the action of the 100,000th and upward; and this it will do and has done a thousand times.

Most persons have more confidence in their own senses than in those of another, more confidence too, perhaps, in what they see than in that they feel. There was a time when men believed on the strength of much less evidence than they do to-day. This is emphatically an age of reason, the evidence furnished by one only of our physical senses scarcely being considered sufficient to form the basis for a reliable belief. Could our patients, in addition to feeling that their pains have disappeared, taste our medicines, as in the other schools of practice, does any one believe we should have so much trouble to convince them of the efficacy of the remedy by which the cure was effected. There can be no doubt that all diseases not mechanical are caused by imponderable particles of matter. Who can weigh the contagion of measles, scarlet fever, or small-pox? Even physicians, whose scales weigh nothing less than a grain, do not hesitate to admit the imperceptible causes of disease.

Baglivi, the Roman Hippocrates, said long ago: "According to Pliny, we are ignorant of what makes us live, but if I dare give my opinion, we are much more ignorant of what makes us sick, for the infinitesimal substance that gives the first and immediate impulse to disease is entirely incomprehensible."

An instance is related by Prof. Boulli, of Turin, where the tooth of a rattlesnake (*Crotalus Horridus*) had been preserved in alcohol for thirty years, and afterward exposed for sixteen years to the weather, after which he punctured with it the skin of an animal, causing its death in one hour. But a fine opportunity was lost in not testing the alcohol in which the tooth had been placed as to its poisonous effect on animal life, as well as the tooth itself; there is little doubt but that it would have been found to have been equally poisonous, though probably defying the finest chemical test.

This experiment would go far to prove that although alcohol may possibly act as an antidote to snake poison after it has entered the circulation, it will not neutralize or destroy it without a medium through which to act, and the question then arises, How do poisons antidote each other? It cannot be by the direct action of the one on the other, else this change would occur out of the system as well as in it. It must be then that the alcohol acts on, or forms a chemical union with, the properties of the blood for which the virus has an affinity, thereby changing the sanguineous fluid to such an extent that the force of the poison is exhausted, as a fire goes out for the lack of more combustible material. We are further led to believe this from the toxical effects of the two poisons. It is well known that alcohol destroys the oxygen of red globules of the blood, generating carbonic acid in its stead, producing that dark, bloated, purple appearance, which is seen in hard drinkers, and there can be little doubt but that snake poison does the same, from the rapid tendency to decomposition, discoloration, and gangrene in cases where death has occurred from it, and it is very probable that it is in this way not only that poisons antidote each other, but that medicines cure diseases. A case is related as having occurred in Italy some thirty years ago, where a man, bitten by a rabid dog and ill of hydrophobia, was repeatedly bitten by the poisonous viper; and although the case terminated fatally, it is said the symptoms were entirely changed, and that he died more from the poison of the snake than from that of the dog—an instance of fatal aggravation, if you like, from too low a dilution too often repeated.

Now, this was homœopathic only so far as the law of similars was concerned, but it illustrates nothing new. For, in a poem published in Sanscrit fifty-six years B. C., the author says, "It has been heard of old time in the world that poison is the remedy for poison." And when we reflect that this was written nearly two thousand years ago, and that the opinion was then said to have been "old in the world," it certainly looks like disputing the right to antiquity with the principle, *contraria contrariis.*

A milligram of mercury in solution in twenty quarts of water will kill fish in a few seconds, and yet this proportion is so small as to defy the most delicate chemical test. Atmosphere containing the one million two hundred and fifty thousandth part of sulphureted hydrogen, inhaled by a horse, will kill him in a few minutes. The animal economy is a strange piece of mechanism, of which far too little is known. Man is usually considered the grandest work in the scale of existences, and yet his anatomy, as wonderful as it is, will bear no comparison with that of the worm he crushes beneath his feet, or the tiny insect that floats like a useless mote in the summer air. In the human subject we find three hundred and seventy muscles, yet Lyonet, who devoted his whole life to the observation of a single species of caterpillar, discovered in it four thousand. A common house-fly is said to have eight thousand eyes, and some butterflies have twenty-two thousand. The motion of a gnat's wing is six hundred thousand times in a minute, but the dimensions of the muscles that impart this rapid motion who can measure, or the size of the innumerable parasites that it is said find a home beneath the fimbria of those wings with ample space to range at will? We are far from knowing everything, far from being able to give an explanation for every phenomena witnessed. If we could do so, there would be little left for the researches of the coming ages. We are not obliged to answer all the objections urged against Homœopathy. Our premises taken, our conclusions are legitimate, and it remains with our opponents to prove them to be incorrect. The smallness of the dose is one of the questions most generally raised, and that, we think, has been pretty fully answered. It is custom and prejudice that makes many so reluctant to admit that it is the quality and adaptation of matter, instead of its quantity, that produces the desired results. And this prejudice is by no means confined to the allopathic school of practice. I doubt if greater skeptics can be found anywhere than are to be met with among so-called homœopathists, men who probably never made a homœopathic cure in their lives, and who, should they witness one, would be astonished beyond measure and as ready as any one to attribute the result to other causes than the medicine. Low potencies and crude drugs suppress symptoms in the same way, and the prescriber who, in his therapeutics, has never risen above them, has never breathed the refined atmosphere of pure Homœopathy. Previous to Hahnemann's time, while many were willing to admit that invisible causes were capable of producing visible effects, so far as causing disease was concerned, no one seemed to conceive that microscopic remedies might also cure.

Because of this widespread skepticism or lack of knowledge, in and out of our ranks, of what really constituted Homœopathy, the charge over and over again has been made by its enemies that all our old landmarks were obliterated, or that we ourselves were beginning to see and to admit that Hahnemann's teaching was all a fallacy. But there is not and never has been any such admission made on the part of his true disciples, and of the future of Homœopathy we have no fears, though it is humiliating to be obliged to admit that misguided fanatics in our ranks, either from ignorance of what constitutes Homœopathy, or from personal ambition for distinction, have led us so near the picket-line of the enemy that some desertions have already occurred, and others are calling for a truce which savors much of compromise. In view of this state of affairs, on the 16th day of June, 1880, we raised the old flag, around which forty years ago battles were fought and victories won. Eighty stalwarts have already rallied to its defense, and the recruiting still goes on. We now have enrolled soldiers from England, Italy, and Spain, and they are coming from every civilized country on earth, not enlisting for one or for two years, but for the war or for a lifetime.

At the first meeting after our organization, owing to the absence of the secretary and treasurer with all the papers, names of members, etc., and without any order of business, and with few by-laws or resolutions by which to be governed, the first year of the existence of the Association might be said to have been well-nigh lost. At the second meeting, the conditions of things was but little better. No officer, except the president, being in attendance—vice-president, secretary, treasurer, and the chairman of every bureau being absent—and with no order of business, it was hard for the president to bring order out of chaos, or to proceed with any system. But many valuable papers were presented, the authors of which, with few exceptions, were not present; these were read and discussed, and new members and new officers, with the exception of the president, were elected for the ensuing year.

Some of the members who were not present thought the re-election of your president was a mistake. Of course, this was a mere matter of opinion. There was no precedent for or against this; we were merely making a precedent, making history, and it is not at all likely that everything we might do would be entirely satisfactory to every member, or anything we might say meet the approbation of those who could not be received as members, and the teaching and practice of whom made the organization of this Association necessary. The idea has been circulated with a good degree of industry by those who would gladly see its dissolution, assisted, as they claim, by one or more disaffected members, that there is a widespread dissatisfaction in our ranks growing out of words spoken by your president at our last meeting, to wit: That if the destructive policy as mapped out by some of the leaders of the American Institute was henceforth to be its governing principle, I could see no good reason why we should not sever our connection with it, and that my re-election after expressing these sentiments amounted to an indorsement of them by the Association. And a great amount of capital was sought to be made on this account. But it is not fair to assume that your president's re-election was in any sense owing to his views on this subject, neither do I believe that it should or would defeat the election to the presidency of any member who entertained similar views. And after a year's reflection your president is not disposed to retract anything he may have said in relation to this subject.

The American Institute was organized, the same as the International Association, to perpetuate and disseminate the inductive methods of Hahnemann. Had it continued to do this, the organization of this Association would have been a superfluity. If any member still believes it is now following its original intent, then his line of duty to him must be clear—merely to follow where it leads and to oppose whatever he may conceive to be antagonistic. So far as I am acquainted with the views of the members of this Association, it is with feelings of deep regret that they conceive the necessity for such an organization exists, and willingly would they see it disband did they believe that the best interests of Homœopathy as Hahnemann taught it would be promoted thereby. But every year the necessity for its existence seems to become more and more apparent, and more and more do we feel sad in view of this fact, for we have not now, neither have we ever, entertained any but the kindest feelings toward the American Institute either individually or collectively, and gladly would we see it, with or without our aid, pause on its road to ruin and return to its first love. But if it will not do this, but take the advice of one of its members, to turn us all out because, he says, we "hang like a ball and chain on the limbs of the Institute, obstructing its progress"—though he does not make it clear whether the disgrace is to be attached to the ball and chain or to the wearer—or follow the lead of another member, and have us placed in a separate apartment or bureau, denying to us even the name of homœopathists;—if this policy is to be adopted we may clearly see that our Association was not organized a day too soon, and any such action on the part of the Institute should serve as a stimulus to every member for renewed efforts to preserve intact that inheritance left us by Hahnemann and his early disciples. There can be no fears that truth can ever die, though a diamond may be dimmed while marble is polished; but if our Association does not prosper and our cause succeed, it will not be because we have not truth on our side, but because we do not defend it with that zeal and energy which is the element of success in any and every undertaking. Every member should feel that he has a duty to perform, a duty that he owes to himself, to Homœopathy, and to humanity, and instead of absenting himself from our meetings, entering objections, and creating dissensions, he should be present to give his counsel in correcting or preventing mistakes.

It has also been urged that our Association has fallen short of accomplishing the end and aim of its organization. But national or international influence is not gained in a day, character and reputation are of slow growth, the education of the people is a great work.

It is impossible to compute the amount of influence in this direction we have already accomplished, not only with the laity, but with physicians as well. Whoever thinks nothing has been done should obtain a report from the pharmaceutists as to the increased demand within the past year or two for high potencies. Physicians prominent in the profession are buying and using them, and honest investigation usually results in honest conversion; and as our paramount object was to give strength and influence to Hahnemannian Homœopathy, we are not discouraged at what three years have accomplished. Besides, the amount of abuse we have within the same time received from the eclectic wing of our school only convinces us more and more of the necessity and justice of our course.

As for your president, he is not sorry that his term of office is nearly closed; it has been attended with more honor, perhaps, than either thanks, pleasure, or profit; but for none of these has he labored, and has not therefore been disappointed. His every act and word in regard to the Association has been, as he supposed, for its best interests. It is not at all to be presumed that every one can see things in the same light, but my endeavor has been as far as possible to avoid petty jealousies and prejudices, and without consulting others as to what language I should use, though with no intention of giving offense to any, I have on all occasions spoken the truth as I understood it, and henceforth will be glad to work for the success of our cause in any capacity that may be assigned me, knowing that there is no excellence without labor, and that if we fail it will not be so much from labor wrongly directed as from no labor at all, or only that which exhibits itself in words. No true Hahnemannian lives that I would not gladly take by the hand and welcome to membership. Homœopathy as represented by this Association is my friend, and if its opposers, or its enemies, receive little sympathy or little mercy at my hands, it is not that I love them less, but that I love Homœopathy more. No man would rejoice more than I to see a union of all schools of medicine, but it would have to be upon the platform as laid down in the *Organon* of Hahnemann. As for a union on any other basis, no homœopathist has ever harbored such a thought. We are much obliged to the medical societies of other schools for their kindness in permitting their members to consult with us, giving as a reason that in doing this it gives us the opportunity of becoming better acquainted with their mode of treatment, which they think we will then be likely to adopt; but they forget that our familiarity with their treatment is one great reason why we are homœopaths. We therefore regard their propositions to meet us in consultation as entirely gratuitous, and presume that with or without their society resolutions, they will in the future, as in the past, always consult with us *whenever they are invited to do so.* And, in conclusion, I can only express the hope that no diversity of opinion about minor matters and no personal difficulty will prevent any and every Hahnemannian, wherever he may be, from joining us, not by any means for the purpose of being antagonistic to any other medical organization, but for the far higher and nobler purposes of an interchange of thought for the welfare of the afflicted, and for the perpetuation of the art of healing as taught by Samuel Hahnemann.

